# Targeted Demethylation of FOXP3-TSDR Enhances the Suppressive Capacity of STAT6-deficient Inducible T Regulatory Cells

**DOI:** 10.1007/s10753-024-02031-4

**Published:** 2024-05-03

**Authors:** Rubén D. Arroyo-Olarte, Juan C. Flores-Castelán, Leonel Armas-López, Galileo Escobedo, Luis I. Terrazas, Federico Ávila-Moreno, Sonia Leon-Cabrera

**Affiliations:** 1 Unidad de Biomedicina. Facultad de Estudios Superiores-Iztacala, Universidad Nacional Autónoma de México, Av. De los Barrios 1, Los Reyes Iztacala, Edo. De México, Tlalnepantla, México; 2 Laboratory of Immunometabolism, Research Division, General Hospital of Mexico “Dr. Eduardo Liceaga”, 06720 Mexico City, Mexico; 3 Laboratorio Nacional en Salud, Facultad de Estudios Superiores-Iztacala, Universidad Nacional Autónoma de México, Edo. De México, Tlalnepantla, México; 4 Carrera de Médico Cirujano, Facultad de Estudios Superiores Iztacala, Universidad Nacional Autónoma de México, Av. De los Barrios 1, Los Reyes Iztacala, Edo. De México, Tlalnepantla, México

**Keywords:** Tregs, CRISPR-Cas9, TET1, DNA methylation, FOXP3, STAT6

## Abstract

*In vitro* induced T regulatory cells (iTregs) are promising for addressing inflammation-driven diseases. However, current protocols for the generation and expansion of iTregs fail to induce extensive demethylation of the Treg-specific demethylated region (TSDR) within the *FOXP3* gene, recognized as the master regulator for regulatory T cells (Tregs). This deficiency results in the rapid loss of Foxp3 expression and an unstable regulatory phenotype. Nevertheless, inhibition of STAT6 signaling effectively stabilizes Foxp3 expression in iTregs. Thus, this study aimed to develop a protocol combining epigenetic editing with STAT6 deficiency to improve iTregs’ ability to maintain stable suppressive function and a functional phenotype. Our findings demonstrate that the combination of STAT6 deficiency (STAT6-/-) with targeted demethylation of the TSDR using a CRISPR-TET1 tool leads to extensive demethylation of *FOXP3*-TSDR. Demethylation in STAT6-/- iTregs was associated with enhanced expression of Foxp3 and suppressive markers such as CTLA-4, PD-1, IL-10, and TGF-β. Furthermore, the edited STAT6-/- iTregs exhibited an increased capacity to suppress CD8+ and CD4+ lymphocytes and could more efficiently impair Th1-signature gene expression compared to conventional iTregs. In conclusion, the deactivation of STAT6 and TSDR-targeted demethylation via CRISPR-TET1 is sufficient to induce iTregs with heightened stability and increased suppressive capacity, offering potential applications against inflammatory and autoimmune diseases*.*

**Supplementary Information:**

The online version contains supplementary material available at 10.1007/s10753-024-02031-4.

## Introduction

Regulatory T cells (Tregs) play a pivotal role in controlling inappropriate immune responses by suppressing immune effector cells. They are crucial for maintaining tolerance to self- and non-self-antigens, ensuring immune homeostasis, and suppressing inflammation [1]. Within the Tregs, two main subpopulations are recognized: natural thymic Tregs (nTregs) and induced peripheral Tregs (iTregs). These populations differ in origin and specific phenotypic markers, with notable distinctions in stability and suppressive capacity.

The long-term and consistent expression of Foxp3 in nTregs and their stable suppressive characteristics are regulated by the demethylation of genes specific to the Treg epigenetic signature. This includes an evolutionarily conserved CpG-rich segment within the *FOXP3* locus known as the Treg-specific demethylated region (TSDR). Successful Foxp3 induction and maintenance are required for immune suppressive function. Human conventional T cells and *in vitro* TGF-β-induced iTregs are susceptible to losing Foxp3 expression and, consequently, iTreg functionality because they do not undergo TSDR-dependent activation through demethylation. In addition, iTregs have been shown to convert into pathogenic Foxp3- cells after losing Foxp3 expression, hindering their capacity to control inflammatory conditions, such as graft vs. host disease [2].

Previously, our research established the crucial role of the signal transducer and activator of transcription 6 (STAT6) as a regulator of Tregs' function both *in vitro* and *in vivo*. In the context of intestinal inflammation, STAT6 knockout (STAT6-/-) mice exhibited elevated levels of CD4+Foxp3+CD25+ cells in the colon, circulation, and spleen, accompanied by increased expressions of TGF-β, IL-10, and Foxp3 compared to their wild-type (WT) counterparts [3]. The heightened abundance of Tregs in a state of STAT6 deficiency was associated with slight intestinal damage, indicating enhanced suppressive activity in inflammatory conditions. Additionally, STAT6-deficient induced regulatory T cells (STAT6-/- iTregs) exhibited prolonged stability and suppressive efficacy *in vitro* and during the early stages of a colitis-associated cancer model. This sustained functionality was linked to a partially demethylated TSDR compared to their WT counterparts [4]. However, the extent of demethylation remained notably lower than that observed in thymic nTregs, raising concerns about the potential for phenotype loss when considering their use in adoptive cell therapy for inflammatory conditions.

The CRISPR-Cas9 system, an adaptive immunity system found in prokaryotes, includes a nuclease (Cas9) whose activity depends on the binding and recognizing of a complementary DNA target sequence by a short 21 nt guide RNA (gRNA). This system has been adapted and expanded as a genetic editor in various organisms [5]. One such adaptation is CRISPR-TET1, which involves the fusion of a catalytically "dead" Cas9 (dCas9) with a demethylase domain (TET1-CD), while preserving the dCas9’s gRNA-dependent DNA binding activity. CRISPR-TET1 has been shown to perform targeted demethylation of human [6] and murine [7] genes, including *FOXP3*. However, these studies have demonstrated only partial transcriptome remodeling towards a Treg signature, indicating that TSDR demethylation alone cannot induce a functional Treg phenotype. Therefore, this study aimed to combine targeted TSDR demethylation via CRISPR-TET1 with STAT6 deficiency to obtain more potent and stable iTreg suppressive capacity.

## Materials and Methods

### Mice

Eight- to ten-week-old wild-type (WT) Foxp3^EGFP^ knock-in mice (B6.Cg-Foxp3tm2Tch/J) and STAT6-/- Foxp3^EGFP^ mice co-expressing Foxp3 and enhanced GFP under the control of the *FOXP3* promoter were generated and maintained as previously reported [4]. BALB/c mice were bred in our animal house and kept in cages according to our institutional guidelines. All experiments were carried out on age- and sex-matched animals. Animal experimentation protocols were approved by the Facultad de Estudios Superiores Iztacala (FES-I) Bioethics Committee for Animal Research.

### Guide RNA Design and Plasmid Cloning

A 20-nucleotide, single-guide RNA (sgRNA), SgTSDR, was specifically designed to target the CpG-rich TSDR region in intron 2 of the murine *FOXP3* gene (see Fig. S1a). Following established protocols, forward and reverse sgRNA oligonucleotides were annealed and initially cloned into the PX458 vector [8]. Subsequently, the sgRNA expression cassette was transferred into the pdCas9-TET1CD-mCherry vector, as detailed elsewhere [7]. The resulting construct, designated as pSgTSDR, facilitates the co-expression of SgTSDR and a fusion protein consisting of a catalytically dead Cas9 (dCas9), TET1 catalytic domain (TET1CD), and mCherry reporter, thereby enabling targeted demethylation of the murine *FOXP3-TSDR* (refer to Fig. S1b). As a negative control, an identical construct containing a catalytically inactive human TET1CD domain (Addgene # 129028) was generated and termed pdTET (see Fig. S1c).

### Cell Culture and iTreg Induction

After sacrifice, mouse spleens were removed, macerated, and treated with a hypotonic ammonium chloride solution to hemolyze erythrocytes. Erythrocyte-depleted spleen cells were layered on top of a discontinuous Percoll™ gradient consisting of 40% and 60% solutions, followed by centrifugation. The cells recovered from the 40–60% Percoll interlayer (comprising lymphocytes and monocytes) were harvested and subsequently washed twice with phosphate-buffered saline (PBS). Next, cells were counted and seeded in 6 mm Petri dishes (2×10^6^ cells/dish) and cultured in Treg medium [RPMI medium (Gibco™) supplemented with 10% (v/v) FBS, non-essential amino acids 1x (Thermo Fisher), 1 mM Sodium Pyruvate (Thermo Fisher), 50 μM 2-Mercaptoethanol (Thermo Fisher), 100 units/mL recombinant mouse IL-2, and 5 ng/mL recombinant human TGF-β (PeproTech)]. The cells were activated for 3 days with 3 μg/mL plate-bound anti-CD3 and anti-CD28 antibodies (BioLegend). Then, cells from the supernatant were harvested, washed with PBS, and either left untreated or transfected with the indicated plasmids.

### Chemical Transfection

A total of 2.0×10^6^ cells were transfected with 2.5 µg of plasmid DNA and TransIT^®^ Jurkat (MirusBio) at a 1:5 ratio (mass/volume), according to the manufacturer’s instructions. Next, cells were cultured for 24 h in Treg medium and taken into culture until analysis.

### Cell Staining and Flow Cytometry

**Fig. 1:**
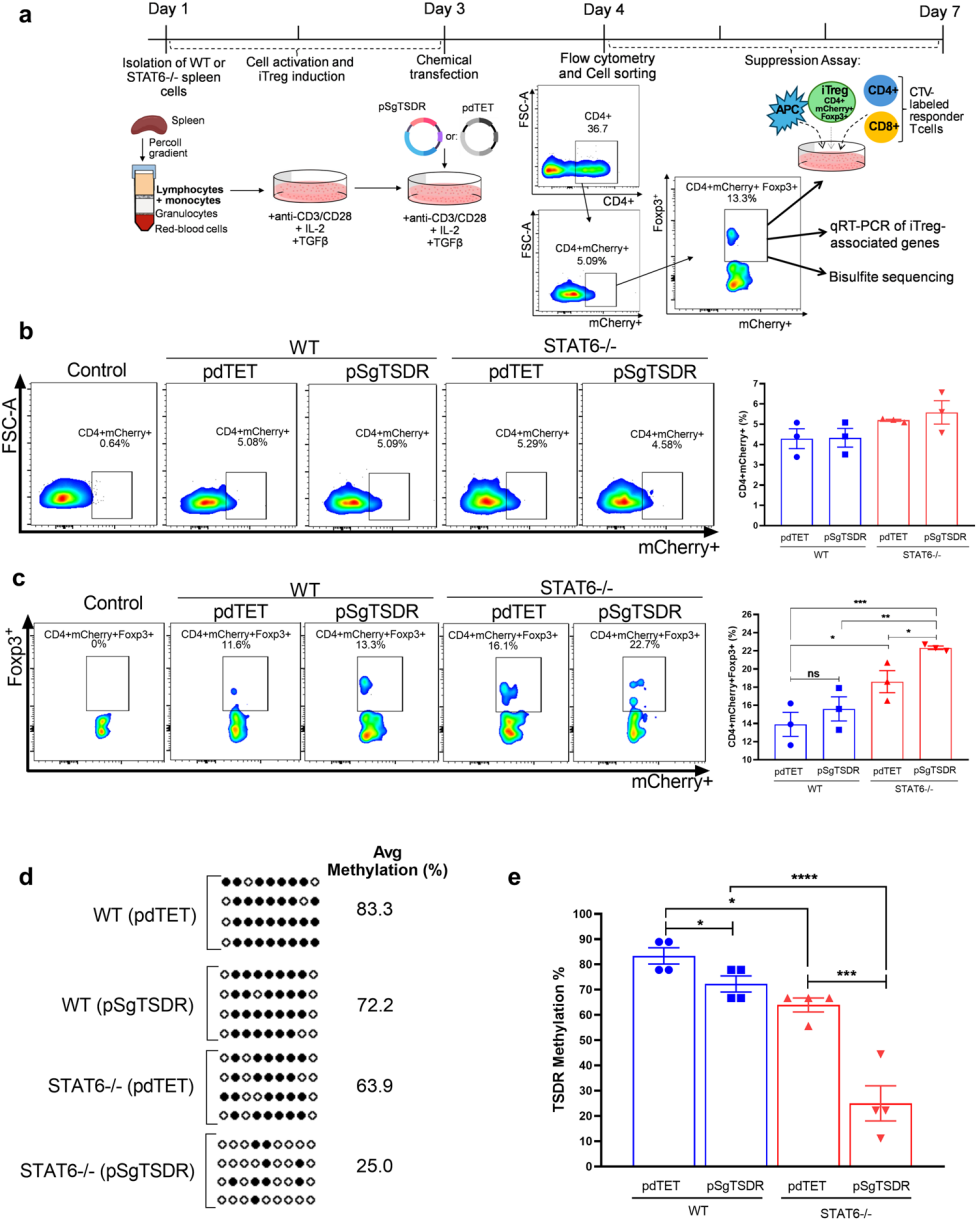
Transfection with a dCas9-TET1 construct leads to targeted TSDR demethylation in iTregs. **a **Workflow for isolation of wild-type Foxp3^EGFP^ (WT) or STAT6-/-Foxp3^EGFP^ (STAT6-/-) splenocytes, iTreg induction, transfection, and gating strategy for FACS cell-sorting of transfected iTregs (CD4+Foxp3+mCherry+ cells) for phenotype analysis using suppression assays, qRT-PCR, and bisulfite sequencing of the *FOXP3*-*TSDR* in each group: pdTET (control-transfected cells) or pSgTSDR (dCas9-TET1-SgTSDR-transfected cells). **b** Representative flow cytometry plots and bar graphs showing the transfection efficiency of pdTET and pSgTSDR within WT and STAT6-/- CD4+ lymphocytes (CD4+mCherry+). Control corresponds to empty-transfected WT splenocytes. **c** Representative flow cytometry plots and bar graphs showing the frequency of iTregs within transfected CD4+ cells (CD4+mCherry+Foxp3+). Control corresponds to pdTET-transfected BALB/c splenocytes (without Foxp3^EGFP^ reporter expression). Data represent the mean ± SEM from three to four experiments with similar results **d **Bisulfite-sequencing analysis of the methylation status of the *FOXP3*-*TSDR* in sorted CD4+mCherry+Foxp3+ cells from each group*.* The degree of TSDR methylation is shown for each of the nine TSDR-CpGs (small circles). Open circles indicate unmethylated, and filled circles indicate methylated CpGs dinucleotides. Total methylation ratios of the entire TSDR were indicated next to the diagrams. **e** Mean methylation degree of the entire TSDR in four samples of each evaluated group. APC: antigen presenting cells, CTV: CellTrace™ Violet staining. *p < 0.05, ***p < 0.001, ****p < 0.0001, ns: non-significant. One-way ANOVA and Bonferroni’s multiple comparison tests were performed.

iTregs were stained with allophycocyanin (APC) anti-mouse CD4 antibody (BioLegend, San Diego, CA, USA) and Zombie Aqua diluted in FACS buffer (Dulbecco's phosphate-buffered saline (DPBS), 1% FBS, and 0.1% sodium azide) at 4 °C for 30 min and washed with PBS. First, dead cells were excluded using Zombie Aqua fluorescence. Then, samples were analyzed based on their mCherry or Foxp3 expression. Samples were acquired on Attune™ NxT (Thermo Fisher, Waltham, MA, USA) or BD LSRFortessa™ Cell Analyzer (BD Biosciences) and data were analyzed with FlowJo vX.10 software (Tree Star, Covington, KE, USA). A minimum of 10,000 live cells were gated and analyzed. For subsequent phenotype analysis, CD4+mCherry+Foxp3+ cells were sorted on a FACSAria (BD Biosciences) Cytometer. The gating strategy is shown in Fig. 1a.

### *In vitro* Suppression Assays

Total splenocytes from Balb/c mice were isolated, incubated with CellTrace™ Violet (ThermoFisher) for 20 min following the manufacturer's instructions, and then seeded onto a 96-well plate pre-treated with 5 μg/mL of anti-mouse CD3 (BioLegend). Transfected iTregs (CD4+mCherry+Foxp3+ cells) were FACS sorted. The isolated iTregs (> 95%) were subsequently seeded into wells with activated responder T cells (Tresp) at different iTreg: Tresp ratios, totaling 100,000 cells per well. The cells were cultured for 3 days at 37 °C with 5% CO_2_ in complete RPMI medium. Cell proliferation was assessed using flow cytometry. Viable cells were gated from the 7-AAD- population and then sub-gated based on CD4+ or CD8+ expression. Peaks for CellTrace™ Violet staining were classified as non-divided or divided cells, and percentages were calculated. CD4+ Tregs were excluded from the analysis due to the absence of CellTrace™ Violet staining.

### RNA Extraction and Quantitative RT-PCR

Total RNA was extracted from 2×10^5^ plasmid-transfected, FACS-sorted iTregs (CD4+mCherry+Foxp3+ cells) 24 h post-transfection using the All-In-One DNA/RNA/Protein Miniprep Kit (BioBasic Markham, ON, Canada) following the manufacturer's instructions. The quality and quantity of total RNA were assessed based on absorbance readings at 260 and 280 nm (A260/A280 > 1.8) using an Epoch™ microplate spectrophotometer (BioTek). Subsequently, 40–100 ng of total RNA was reverse transcribed using the RevertAid first-strand cDNA synthesis kit (Thermo Fisher) following the manufacturer's instructions. A 1 μL aliquot of cDNA was then subjected to real-time quantitative PCR in a CFX 96-well one-touch real-time PCR system™ (Bio-Rad, Hercules, CA, USA) using the SYBR Green qPCR Master Mix (MedChem Express) and gene-specific oligonucleotides listed in Table S1. mRNA expression values were calculated using the 2^−ΔCT^ method with 18S as the housekeeping gene. The obtained values were normalized as the fraction of the average expression in the pdTET-transfected wild-type (WT) group for each evaluated gene.

### Bisulfite Sequencing

Bisulfite sequencing was employed to assess the methylation status of the Treg-specific demethylated region (TSDR) of the *FOXP3* gene in plasmid-transfected, FACS-sorted iTregs (CD4+mCherry+Foxp3+ cells) 24 h post-transfection. Genomic DNA (gDNA) was extracted from 1–2×10^5^ cells using the DNeasy Blood & Tissue Kit (Qiagen, Hilden, Germany). Subsequently, sodium bisulfite modification of gDNA was carried out using the EpiJet Bisulfite Kit (Thermo Fisher) following the manufacturer's instructions. The quality and quantity of both untreated and bisulfite-treated gDNA were assessed based on absorbance readings at 260 and 280 nm (A260/A280 > 1.8) using an Epoch™ microplate spectrophotometer (BioTek). Bisulfite-treated DNA was PCR amplified using the following primers: 5’-GGGTTTTTTTGGTATTTAAGAAAGAT-3’ and 5’-AAATCTACATCTAAACCCTATTATCACA-3’ that recognized *FOXP3*-*TSDR* (Fig. S1). The PCR protocol included an initial denaturation step at 94 °C for 3 min, followed by 40 cycles of denaturation at 94 °C for 15 s, annealing at 55 °C for 30 s, and extension at 72 °C for 30 s. The PCR products were visualized on 2% agarose gels and purified using the PureLink Quick Gel Extraction PCR Purification Combo Kit (Thermo Fisher). Purified PCR products were Sanger-sequenced in the Applied Biosystems 3730xl Genetic Analyzer sequencer using primers specifically recognizing the FOXP3/TSDR region (Fig. S1, Table S1). Four sequences were obtained per each experimental group and were aligned and analyzed for modified (C → T, demethylated) and unmodified (methylated) CpG sites using the QUMA (QUantification tool for Methylation Analysis) web platform suite [9].

### Statistical Analysis

The statistical analysis was performed using GraphPad Prism 9.5 software (GraphPad Software, San Diego, CA). Differences between more than two groups were calculated using the one-way ANOVA test and Bonferroni's multiple comparison tests. All data are presented as mean ± Standard error (SEM). p < 0.05 was considered significant.

## Results

### Transfection with a dCas9-TET1 Construct Leads to Targeted TSDR Demethylation in Wild-type and STAT6-deficient iTregs

The workflow for T cell isolation, activation, iTreg induction, transfection, and gating strategy is illustrated in Fig. 1a. As an initial readout, 24 h post-transfection, cells were gated on CD4+ and analyzed for mCherry expression. In all groups, approximately 4-5% of cells were mCherry+ , indicating successful transfection with either pSgTSDR (construct for targeted demethylation of the murine *FOXP3*-TSDR) or pdTET (negative control) (Fig. 1b). To examine the impact of dCas9-TET1-mediated TSDR demethylation, we analyzed Foxp3 expression within transfected CD4+ cells (CD4+mCherry+Foxp3+). As previously reported [4], STAT6 deficiency led to an elevated iTreg frequency compared to wild-type counterparts, independent of the transfected plasmid. However, pSgTSDR transfection further increased iTreg frequency, particularly in the STAT6-/- group compared to pdTET-transfected cells, suggesting an activation of Foxp3 expression by TSDR demethylation (Fig. 1b). Total demethylation of the intronic TSDR of the *FOXP3* gene has been demonstrated in thymic Tregs from murine and human origin and has been shown to be essential for regulation and stability of Foxp3 expression [10]. To assess whether transfection with the dCas9-TET1 construct demethylates the *FOXP3*-*TSDR*, we analyzed the DNA methylation status of transfected iTreg (CD4+Foxp3+mCherry+) cells through bisulfite sequencing (Fig. 1d, e). Our results show that extensive demethylation of the *FOXP3*-TSDR (75% of analyzed CpG islands) was only achieved in pSgTSDR-transfected STAT6-/- iTregs (75%), compared to pdTET-transfected STAT6-/- iTregs (36%), pSgTSDR-transfected WT iTregs (28%), and pdTET-transfected WT iTregs (17%).

Additionally, mRNA expression of DNA methyltransferases was evaluated. DNMT1A mRNA levels were decreased in WT and STAT6-/- pSgTSDR-transfected iTregs, compared to control pdTET-transfected cells, which could contribute to their lower TSDR methylation status (Fig. S2A). DNMT3A mRNA levels, on the other hand, were unaltered in all groups except in STAT6-/- pSgTSDR-transfected iTregs, which showed a ∼2-fold increase compared to control pdTET-transfected cells (Fig. S2b).

### dCas9-TET1-mediated TSDR Demethylation Increases iTreg Frequency and mRNA Expression of Foxp3 and Treg-associated Genes in STAT6-/- CD4+ cells

**Fig. 2:**
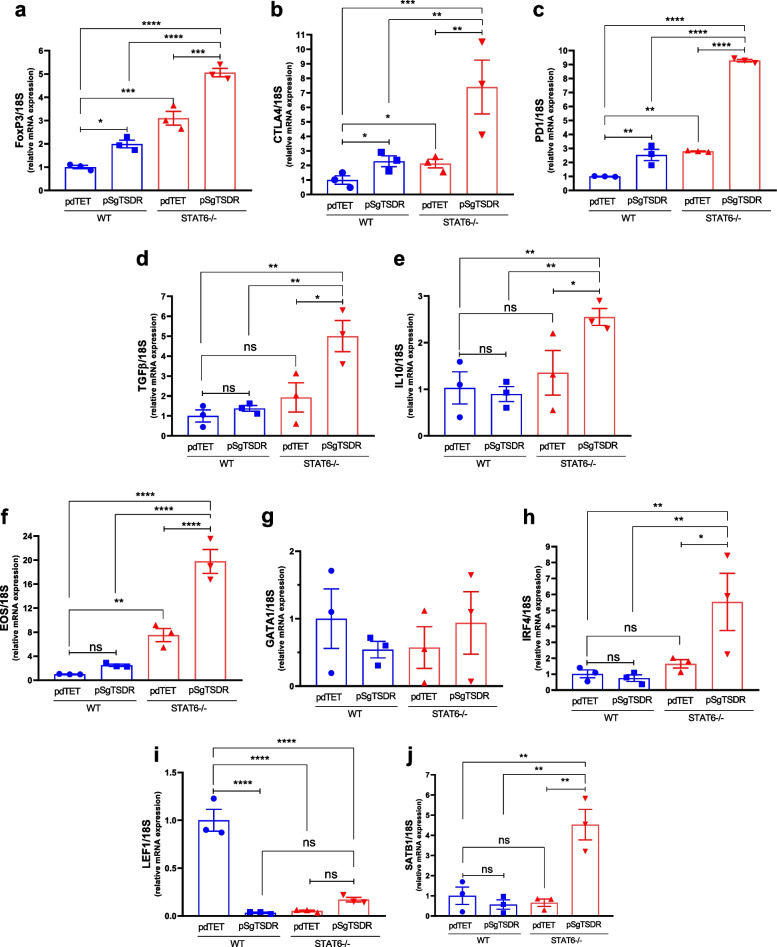
The targeted TSDR demethylation mediated by dCas9-TET1 in both WT and STAT6-/- iTregs influenced the expression of Treg suppression markers*.* **a-e** mRNA expression levels of Treg phenotype markers: **a** FOXP3, **b** CTLA-4, **c** PD-1, **d** TGF-β1, and **e** IL-10. **f–h** Foxp3 transcription factor partners in CD4+mCherry+Foxp3+ FACS-sorted cells for pdTET (inactive plasmid-transfected cells) or pSgTSDR (dCas9-TET1-SgTSDR-transfected cells): **f** EOS, **g** GATA1, **h** IRF4, **i** LEF1, and **j** SATB1. Data represent the mean ± SEM from three experiments with similar results. *p < 0.05, **p < 0.01, ***p < 0.001, ****p < 0.0001, ns: not significant. One-way ANOVA and Bonferroni's multiple comparison tests were performed.

We then evaluated whether the dCas9-TET1-mediated, targeted TSDR demethylation induced the mRNA expression of Foxp3 and genes associated with the Treg phenotype. A notable, 2-fold elevation in Foxp3 mRNA was observed in WT iTregs transfected with pSgTSDR compared to pdTET-transfected cells (Fig. 2a). pdTET-transfected STAT6-/- iTregs also showed increased Foxp3 mRNA levels, by a factor of 3, compared to control pdTET-transfected WT iTregs, and this was enhanced to a 5-fold increase upon pSgTSDR-transfection (Fig. 2c). The mRNA levels of the suppressive markers CTLA-4 (Fig. 2b) and PD-1 (Fig. 2c) exhibited a consistent pattern. Both pSgTSDR-transfected WT and pdTET-transfected STAT6-/- iTregs displayed an approximately 2-fold increase in these markers. Interestingly, pSgTSDR-transfected STAT6-/- cells demonstrated a more substantial rise, reaching up to a 7-9-fold increase in both markers compared to control pdTET-transfected WT iTregs. The mRNA expression levels of the cytokines TGF-β and IL-10 were assessed. pSgTSDR transfection exhibited no discernible impact on WT iTregs. However, pSgTSDR STAT6-/- transfected cells exhibited a noteworthy upswing in the mRNA levels of TGF-β (approximately 5-fold, Fig. 2d) and IL-10 (2.5-fold, Fig. 2e). While *FOXP3* is essential for the specification and maintenance of Tregs and is considered the 'master regulator' of Tregs, a number of other transcription factors (TFs) have been reported to interact with *FOXP3* and promote Treg function. These include TFs such as EOS, IRF4, SATB1, LEF1, and GATA1 [11]. We analyzed mRNA levels in CD4+mCherry+Foxp3+ FACS-sorted cells after transfection. The mRNA expression of EOS was upregulated 2-fold in STAT6-/- pdTET-control-transfected iTregs, and this upregulation was further enhanced to a 5-fold increase upon pSgTSDR transfection compared to WT transfected cells (Fig 2f). Conversely, no significant changes were observed in GATA1 mRNA expression (Fig. 2g). The mRNA expression levels of IRF4 and SATB1 were enhanced 4-5-fold only in the pSgTSDR-transfected STAT6-/- iTregs (Fig. 2h, j). On the other hand, LEF1 mRNA was significantly downregulated in all conditions compared to pdTET-transfected WT iTregs (Fig. 2i).

### The dCas9-TET1-mediated Targeted Demethylation of TSDR in Both WT and STAT6-/- iTregs Impairs Th1-signature Genes

**Fig. 3:**
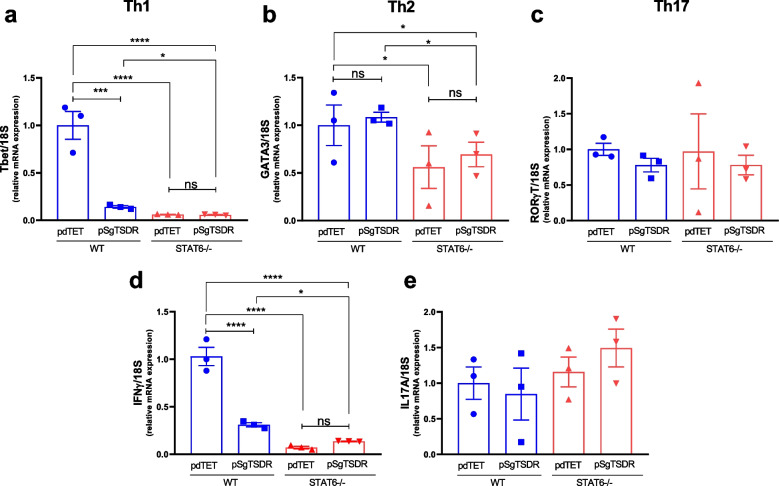
mRNA expression levels of Th-signature genes in CD4+mCherry+Foxp3+ FACS-sorted cells after dCas9-TET1-mediated targeted demethylation*.* mRNA expression of **a** T-bet, **b** GATA-3, **c** RORγ-T, **d** IFN-γ, and **e** IL-17A in CD4+mCherry+Foxp3+ FACS-sorted cells after transfection with pdTET (control-transfected cells) or pSgTSDR (dCas9-TET1-SgTSDR-transfected cells). Data represent the mean ± SEM from three experiments with similar results. *p < 0.05, ****p < 0.0001, ns: not significant. One-way ANOVA and Bonferroni's multiple comparison tests were performed.

To analyze whether dCas9-TET1-mediated targeted TSDR demethylation could also affect the expression of the master regulators of other T helper (Th) cell lineages (Th1, Th2, and Th17), we measure mRNA expression of T-bet, GATA-3, and RORγ-T in iTregs after transfection (Fig. 3a-d). The master regulator of Th1 Cells, T-bet, showed an inverse association with TSDR demethylation in WT iTregs. pSgTSDR-transfected WT iTregs presented an 80% reduction in T-bet mRNA levels compared to control pdTET-transfected WT iTreg cells (Fig. 3a). Notably, STAT6-/- iTregs exhibited a remarkable 90% decrease in T-bet mRNA levels, regardless of the transfected construct. This suggests that the deficiency in STAT6 alone leads to hampered T-bet expression in iTregs (Fig. 3a). The mRNA expression of GATA-3, a transcription factor that drives the differentiation of Th2 cells, was not affected by pSgTSDR transfection. As expected, the expression of the Th2 master regulator GATA-3 decreased 50% in STAT6 deficient cells compared to WT iTreg cells (Fig. 3b). The mRNA levels of the Th17 cell master transcription regulator RORγ-T showed no alterations in any of the groups (Fig. 3c). The mRNA expression levels of Th1 and Th17 downstream cytokines, IFN-γ and IL-17A, were assessed . In pSgTSDR-transfected WT iTregs, IFN-γ mRNA levels were significantly reduced to 25%. Notably, STAT6-/- iTregs also exhibited a substantial decrease in IFN-γ mRNA (down to 10–15%), regardless of the transfected plasmid (Fig. 3d). No significant change was observed in IL-17A mRNA expression levels in any group compared to the control pdTET-transfected WT cells (Fig. 3e).

### Targeted TSDR Demethylation Enhances the Suppressive Capacity of STAT6-/- iTregs

**Fig. 4:**
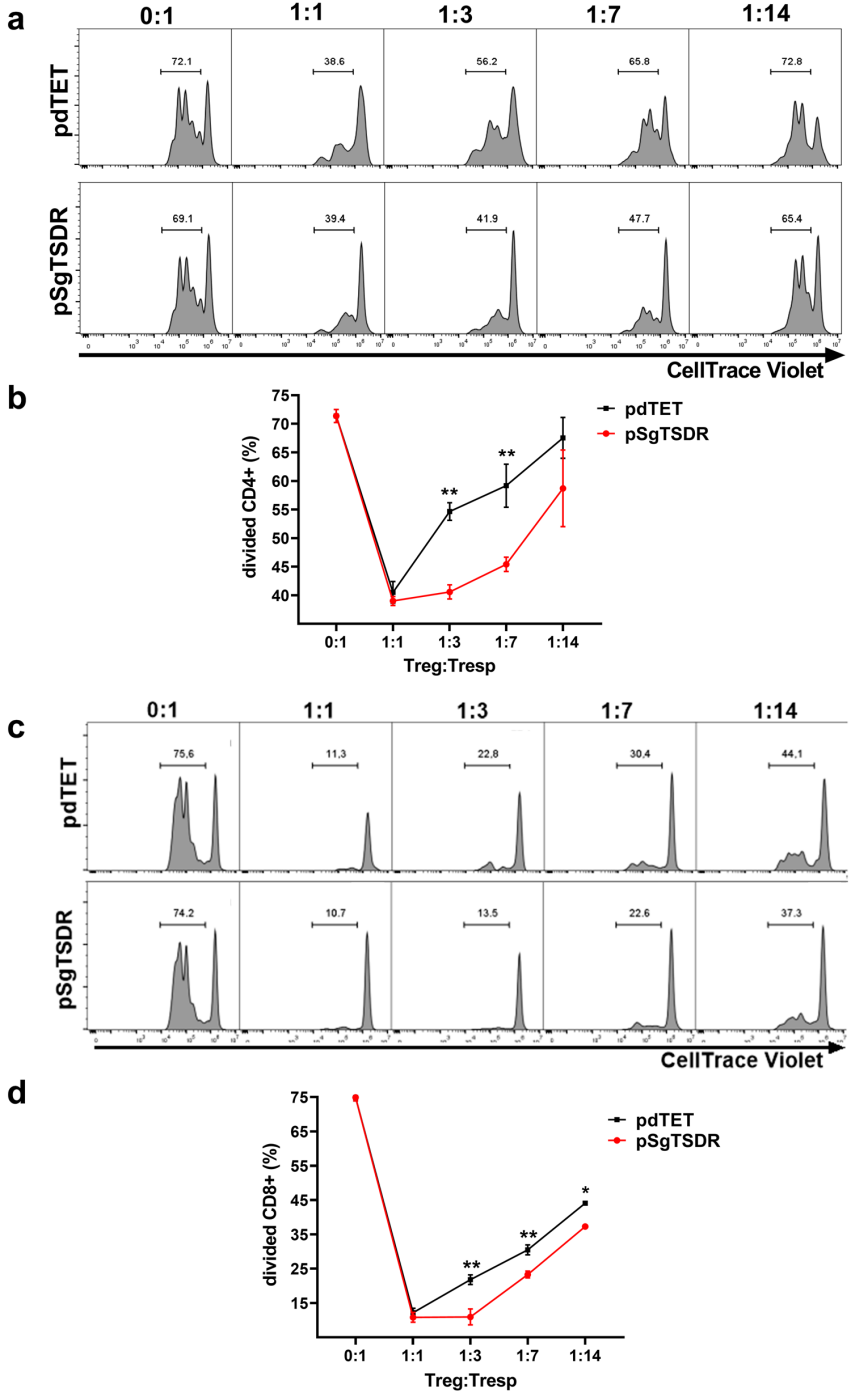
STAT6-/- iTregs with targeted demethylation of TSDR suppress proliferation of CD4+ and CD8+ cells more efficiently. STAT6-/- iTregs were obtained from FACS cell-sorting of transfected (CD4+Foxp3+mCherry+) cells into two groups: pdTET (control-transfected cells) or pSgTSDR (dCas9-TET1-SgTSDR-transfected cells). Activated responder T cells (Tresps) were treated as described in the materials and methods. Histograms show the proliferation of CD4+ (**a**) or CD8+ (**c**) cells in the absence or the presence of STAT6-/- iTregs for 72 h. The numbers indicate the percentage of proliferation with different proportions of iTregs: Tresp cells. Graphs show the percentage of total divided CD4+ (**b**) or CD8+ (**d**) cells with different proportions of iTregs/Tresps. The lymphocyte region was first defined by forward scatter (FSC) and side scatter (SSC) characteristics and further subgated based on CD4 or CD8 expression. Five thousand events from either subgate were captured. Graphs (**b, d**) show the mean ± SEM from three experiments with similar results. *p < 0.05, **p < 0.01. One-way ANOVA and Bonferroni's multiple comparison tests were performed.

Given the substantial alterations observed in Foxp3 expression and mRNA levels of Treg-associated genes in STAT6-/- iTregs following targeted TSDR demethylation, suppression assays were conducted to assess whether these changes could be associated with a superior suppressive capacity. We evaluated the suppressive capability of STAT6-/- iTregs, transfected with pdTET and pSgTSDR, sorted from cultures, against naïve splenocytes stimulated with α-CD3 at varying ratios of iTregs. Upon α-CD3 stimulation of total splenocytes, both CD4+ and CD8+ T cells exhibited robust proliferation, as expected (Fig. 4a and c). Notably, iTregs derived from pSgTSDR-transfected STAT6-/- cultures demonstrated an enhanced suppressive capacity over CD4+ and CD8+ cells compared to control pdTET-transfected iTregs at higher dilutions (1:3, 1:7, and 1:14) (Fig. 4a–d). Collectively, these findings suggest that TSDR demethylation positively influences the suppressive function of STAT6-/- iTregs.

## Discussion

Due to their unstable phenotype, iTregs are not currently considered for use in adoptive T cell therapy despite their potential advantages, such as high scalability [12]. Exploring agents that induce Foxp3 and those that stabilize its expression is necessary to enhance the stability of iTregs.

Previously, we reported that STAT6 deficiency in iTregs preserved a stable phenotype and caused the cells to express high levels of Foxp3 and CD25 during long expansion periods, even in the presence of proinflammatory cytokines such as IL-6 [4], which is known to trigger Treg instability and Th17-like phenotype [13]. Furthermore, we observed that iTregs generated under STAT6 deficient conditions exhibited an elevated demethylation status for the *FOXP3* TSDR, accompanied by reduced mRNA expression of DNA methyltransferase 1 (DNMT1) [4]. This observation implies that STAT6 might contribute to the silencing of the *FOXP3* gene via DNMT1 recruitment [14]. Our previous results showed that STAT6 deficiency in naïve CD4+ T cells promoted the permanence of Foxp3 expression and suppressive function after iTreg differentiation for up to 15 days of expansion. In this study, we searched for the optimal way to generate stable iTregs using STAT6 deficiency with targeted demethylation of the TSDR with a CRISPR-TET1 tool. Epigenetic editing of the TSDR in STAT6-/- iTregs resulted in a significant increase in the expression of Foxp3. In addition, the mRNA expression of suppressive markers such as CTLA-4, PD-1, IL-10, and TGF-β increased.

Previous reports using the CRISPR-Cas9-based method for targeted demethylation of the *FOXP3-TSDR* demonstrated that epigenetic editing resulted in Foxp3 expression. However, epigenetic TSDR modification could not induce a functional Treg phenotype [7]. This is the first report that demonstrates a protocol where the combination of epigenetic editing with STAT6 deficiency improves the expression of Foxp3 and the classical markers for Treg identification, indicating that the RNA expression profile is switched to a Treg signature. While TSDR demethylation alone may not be adequate to induce functional Tregs, simultaneous STAT6 deficiency can enhance functional alterations, as evidenced by the increased capacity to suppress CD4 and CD8 T lymphocytes observed in transfected STAT6-/- iTregs. STAT6 deficiency could also have other Treg-stabilizing effects such as inhibition of the IL-4 mediated conversion of iTregs to Th2-like effector T cells that typically occurs in allergy settings [15]. However, in this study, we did not analyze whether STAT6 deficiency, in conjunction with targeted demethylation of the TSDR, could be detrimental to cells in the long term. Previously, we demonstrated that in an *in vivo* model of colitis-associated cancer (CAC), characterized by inflammatory conditions, significant increases in the number of CD4+CD25+Foxp3+ cells were observed in the colon, circulation, and spleen when STAT6 was absent. These increases were accompanied by overexpression of TGF-β, IL-10, and Foxp3 compared to WT mice [4]. Therefore, at least *in vivo*, the loss of STAT6 improves the stability of Tregs. It would be intriguing to analyze the *in vitro* culture over longer periods of time to verify the viability of STAT6-/- iTregs within pSgTSDR-transfected cells, as well as to confirm their stability after being transferred *in vivo*.

IL-4 signaling through the IL-4R in naive CD4+ T cells leads to the phosphorylation and activation of STAT6. Subsequently, STAT6 translocates into the nucleus, initiating the expression of IL-4-responsive genes driving Th2 differentiation [16]. Several investigations have suggested the close relationship between Tregs and Th2 cells [17]. In the scurfy (Sf) mutant mouse, characterized by the absence of functional Foxp3, Tregs lose their *in vitro* suppressive capacity and exhibit elevated production of Th2-type cytokines. This is further indicated by an increased expression of GATA-3, implying a degree of plasticity between Th2 cells and iTregs [18]. Th2 cytokines IL-4/13 that use STAT6 signaling inhibit the immunosuppression and tolerance induced by Foxp3+ Tregs [19]. Indeed, IL-4 and GATA-3 hamper the *in vitro* differentiation of naive CD4+ T cells into Foxp3+ Tregs in the presence of TGF-β, diminishing their capacity to suppress T cell proliferation. Conversely, Foxp3 can bind to GATA-3, inhibiting the expression of IL-5 and impeding Th2 differentiation [20]. Previous reports have highlighted the antagonistic interaction between STAT6 and Foxp3. Th2 development plays a significant role in the decline in iTreg viability during prolonged culture, primarily due to the direct binding of STAT6 to the *FOXP3* promoter [21]. In accordance with previous results [22], this study showed that STAT6 deficiency significantly decreased GATA-3 expression in iTregs, impairing the Th2 response development which could enhance Tregs` differentiation. Previously, we demonstrated that during *in vitro* Treg expansion (day 15), the secretion of IL-4 increased in cultures from WT cells, contributing to iTreg fragility [4]. In a mouse model, Tregs expressing reduced Foxp3 levels resulted in the onset of a severe autoimmune syndrome, where these cells tend to adopt a Th2-type effector phenotype, even in a Th1-polarizing environment [23]. In addition, *in vitro* iTreg induction diminished the expression of Foxp3 and caused Tregs to revert to effector T cells (especially Th2-like cells), which produce cytokines such as IL-2 and IL-4 [24]. Therefore, modifying the STAT6 signaling axis could be an excellent strategy for improving iTreg cell generation.

T-bet is a transcription factor critical for Th1 differentiation and directly regulates the expression of IFNℽ [25]. T-bet blocks the differentiation of other CD4+ Th cell subsets. Mainly, T-bet binds to the evolutionarily conserved region (ECR) upstream of the *FOXP3* promoter site, blocking its expression [26]. In iTregs with epigenetic editing, we observed a decrease in T-bet and IFNℽ mRNA expression, which correlated with higher Foxp3 expression, compared to control cells. Neuropilin-1 (Nrp-1)+ iTregs have enhanced suppressive function and stability compared to their Nrp-1- counterpart both *in vivo* and *in vitro* [27]. Nrp-1-/- Tregs exhibit elevated T-bet expression and IFN-γ production, resulting in increased Treg fragility [28]. While, in this study, we did not specifically analyze the expression of Nrp1, the reduction in T-bet and IFNℽ expression suggests a potentially more stable phenotype for Tregs.

Multiple redundancies exist in the Treg switch. Some studies have reported the ability of transcription factors (TFs) such as Eos, Irf4, Satb1, Lef1, and Gata1 to flip the switch [11]. Co-immunoprecipitation experiments in transduced Tregs showed interactions between Foxp3 and Gata1, Satb1, and Lef1, suggesting that combining these TFs with Foxp3 could induce the Treg signature [11]. Our experiments detected a significant increase in EOS, IRF4, and SATB1 expression, particularly in pSgTSDR-transfected STAT6-*/-* iTregs. The Treg signature, along with its regulatory elements, is structured with regulatory feedback loops, both positive and negative. This organization allows for the potential to self-assemble and stabilize once the expression of Foxp3 and specific cofactors surpasses that of Tconv cells. Interestingly, in our system, the differentiation of Tregs triggered, directly or indirectly, the transient expression of Foxp3 and one of its cofactors. In the future, it would be interesting to analyze how these cofactors affect the localization of Foxp3 throughout the genome.

Retroviral expression of Foxp3 has been reported to increase CTLA-4 expression, conferring a suppressive function to conventional T cells [29]. However, previous reports using dCas9-TET1 constructs targeted to FOXP3-TSDR failed to induce CTLA-4 expression despite successful TSDR demethylation [7]. In a prior study [6], dCas9-TET1 mediated demethylation of the human TSDR in Jurkat cells. The results indicated augmented Foxp3 expression; however, no substantial alterations were noted in other master regulators associated with various T-helper lineages, such as RORγT, GATA-3, and Tbet. This lack of significant changes suggests that there was no complete commitment to the Treg lineage. In this study, we demonstrated that the synergy between TSDR-mediated Foxp3 induction and STAT6 deficiency not only significantly enhanced Foxp3 expression but also induced the expression of CTLA-4 and PD-1. However, CTLA-4 proteins in Tregs are not stably expressed on the cell surface but are rapidly cycling between the cell surface and the cell interior [30]. One limitation of this study, stemming from the small number of cells obtained post-transfection, was the challenge in assessing extended culture times and confirming the stability of the transfected iTregs and the CTLA-4 expression. Therefore, it would be valuable to explore the tool described here in conjunction with other iTreg-induction protocols that have been previously reported, such as *in vitro* retinoic acid treatment with TGF-β1 that reduces STAT6 binding to the Foxp3 promoter and enhances histone acetylation [21]. In addition, utilizing antagonistic agents to neutralize IL-4 or diminishing STAT6 binding to the Foxp3 promoter could represent novel strategies to enhance the generation of inducible Treg cells and promote tolerance. The efficacy of Ruxolitinib (RUX), a JAK 1/2 inhibitor, in promoting the expansion of Tregs has been studied [31]. Although RUX expands Tregs and impedes the differentiation of CD4+ T cells into IFN‐γ‐ and IL‐17A‐producing cells, its effects are immune-context dependent [32]. In addition, RUX decreases the phosphorylation of STAT5, a transcription factor essential for Foxp3 transcriptional regulation. Thus, the use of specific inhibitors for STAT6 could be a more appropriate strategy.

The utilization of alloantigen-specific regulatory T cells (^allo^Tregs) through adoptive transfer has emerged as a potential immunotherapeutic strategy for kidney transplantation. These expanded ^allo^Tregs have demonstrated effective suppression of T cell proliferation. Nevertheless, prolonged expansion has been associated with heightened methylation of the TSDR [33]. To address this challenge, a novel approach involving epigenetic editing through CRISPR-dCas9-TET1 and the inhibition of STAT6 signaling could offer a promising avenue to prevent the loss of Foxp3 expression during extensive *in vitro* expansion of Tregs.

## Conclusion

The results of this study significantly enhance our understanding of STAT6 signaling function in Tregs and identify the functional consequences of TSDR demethylation and STAT6 deficiency during iTreg differentiation. Targeted DNA demethylation via CRISPR-dCas9-TET1 enables precise control of gene expression within the physiological boundaries of the regulated gene, considering the physiological network of transcription factors. This enables the development of iTreg differentiation protocols suitable for gain-of-function testing, as opposed to approaches involving ectopic overexpression. This marks a crucial advancement in optimizing therapeutic T cell products for adoptive T cell therapy.

## Supplementary Information

Below is the link to the electronic supplementary material.

Supplementary file1 (PDF 465 KB)

Supplementary file2 (PDF 83 KB)

Supplementary file3 (DOCX 26 KB)

## Data Availability

No datasets were generated or analysed during the current study.

## Abbreviations

CNS1: Conserved non-coding sequence 1
CNS2: Conserved non-coding sequence 2
CRISPR: Clustered Regularly Interspaced Short Palindromic Repeats
CTLA-4: Cytotoxic T-lymphocyte-associated protein 4
DNMT1: DNA methyltransferase 1
DNMT3a: DNA methyltransferase 3a
FOXP3: Forkhead boxhead protein 3
GATA1: GATA-binding factor 1
IFN-γ: Interferon gamma
iTreg: Inducible T regulatory cell
nTreg: Natural T regulatory cell
PD-1: Programmed death-1
RORγ-T: Retinoic acid related orphan receptor gamma T
qRT-PCR: Quantitative reverse transcriptase-PCR
STAT6: Signal transducer and activator of transcription 6
TGF-β: Tumor growth factor beta
Th1, Th, Th2, Th17: T helper lineages 1, 2, 17
T-bet: T-box expressed in T cells.
TSDR: Treg-specific demethylated region
