# Supplementary material for: Targeted Demethylation of FOXP3-TSDR Enhances the Suppressive Capacity of STAT6-deficient Inducible T Regulatory Cells
Source: Inflammation. 2024 May 3;47(6):2159–72. doi: 10.1007/s10753-024-02031-4 (PMC11606997; doi:10.1007/s10753-024-02031-4)
Supplement: Supplementary file 1 — Supplementary file1 (PDF 465 KB) [file 10753_2024_2031_MOESM1_ESM.pdf]

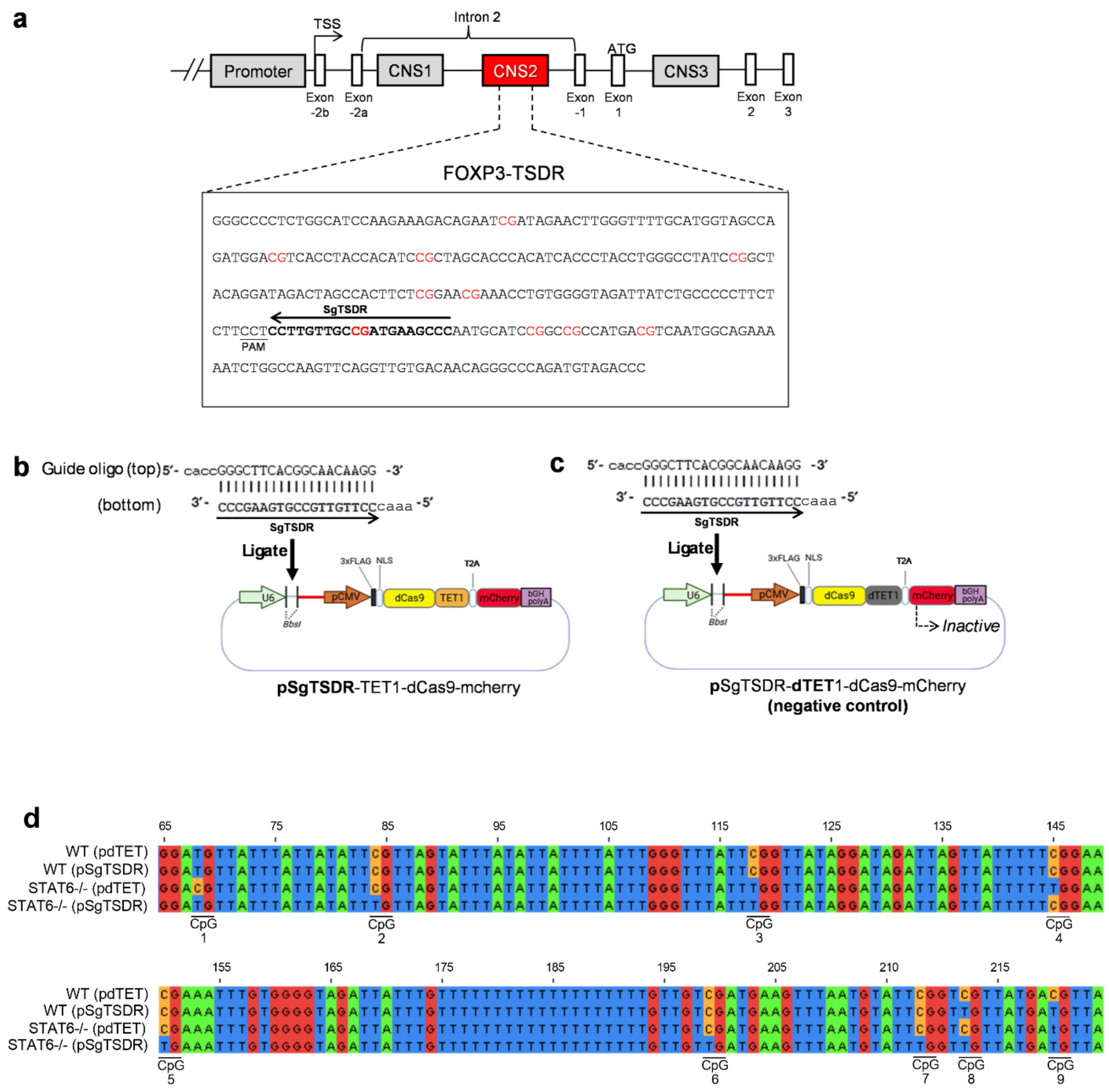

Fig S1. Guide RNA design and plasmid cloning for targeted TSDR demethylation. **a**. Murine *FOXP3* gene architecture and location of guide RNA (SgTSDR) target sequence. Red font indicates CpG islands within the TSDR region. **b-c**. Cloning strategy and plasmid maps for targeted demethylation (pSgTSDR-TET1-dCas9-TET1-T2A-mCherry, pSgTSDR) or negative control transfection (pSgTSDR-dCas9-dTET1-T2A-mCherry, pdTET). The position of the region binding to the guide RNA (SgTSDR) is shown in the reverse strand of the *FOXP3-TSDR*. **d.** Alignment of representative sequences of the *FOXP3-TSDR* after bisulfite treatment for each analyzed condition in this study. Bisulfite treatment converts all cytosines (C) to thymines (T), except those that are methylated, which remain as cytosines. CpG island positions are indicated. CNS1,2,3: Conserved non-coding sequences 1, 2, 3. dTET: catalytically "dead" TET1 domain. FOXP3-TSDR: Treg-specific demethylated region of *FOXP3* gene. T2A: 2A self-cleaving peptide from *Thoseaasigna* virus. TET1: Ten-eleven translocation methylcytosine dioxygenase 1. TSS: Transcriptional start site.
