# Supplementary material for: Targeted Demethylation of FOXP3-TSDR Enhances the Suppressive Capacity of STAT6-deficient Inducible T Regulatory Cells
Source: Inflammation. 2024 May 3;47(6):2159–72. doi: 10.1007/s10753-024-02031-4 (PMC11606997; doi:10.1007/s10753-024-02031-4)
Supplement: Supplementary file 2 — Supplementary file2 (PDF 83 KB) [file 10753_2024_2031_MOESM2_ESM.pdf]

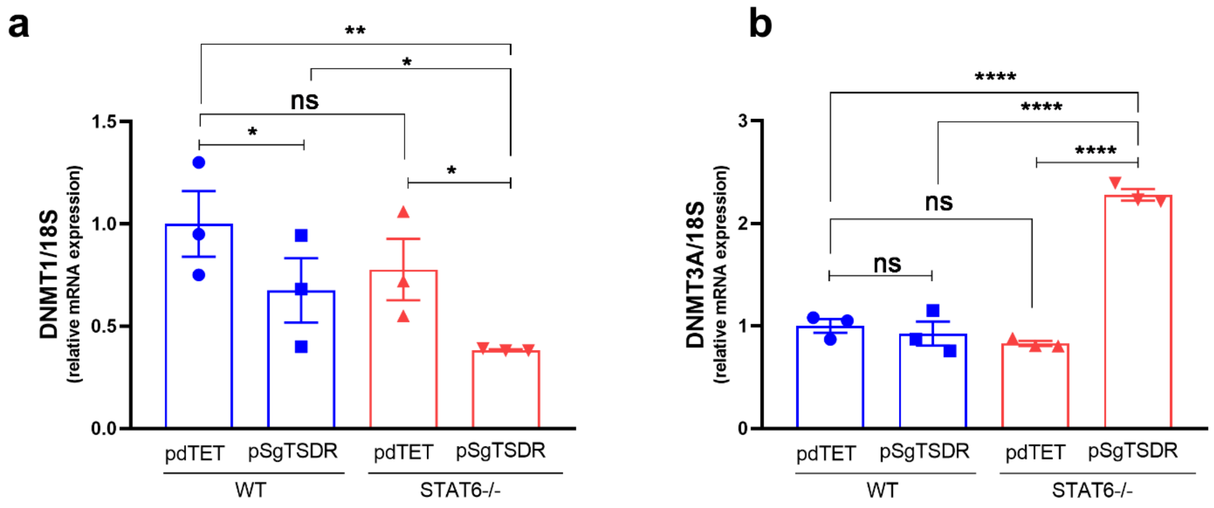

Fig S2. mRNA expression levels of DNA methyltransferases, DNMT1 (**a**) and DNMT3a (**b**), are altered by dCas9-TET1-mediated TSDR demethylation.
