# Supplementary material for: Targeted Demethylation of FOXP3-TSDR Enhances the Suppressive Capacity of STAT6-deficient Inducible T Regulatory Cells
Source: Inflammation. 2024 May 3;47(6):2159–72. doi: 10.1007/s10753-024-02031-4 (PMC11606997; doi:10.1007/s10753-024-02031-4)
Supplement: Supplementary file 3 — Supplementary file3 (DOCX 26 KB) [file 10753_2024_2031_MOESM3_ESM.docx]

**Table S1**. Oligonucleotides used in this study.

| **Name** | **Sequence (5' → 3')** | **Tann (ºC)** | **Function** |
| --- | --- | --- | --- |
| SgTSDR-F | caacGGGCTTCACGGCAACAAGG | 61 | Cloning of guide RNA targeting murine FOXP3-TSDR |
| SgTSDR-R | aaacCCTTGTTGCCGATGAAGCCC | 61 |  |
| Bis-TSDR-F | GGGTTTTTTTGGTATTTAAGAAAGAT | 55 | Amplification of bisulfite-treated gDNA at TSDR for  methylation analysis |
| Bis-TSDR-R | AAATCTACATCTAAACCCTATTATCACA |  |  |
| FOXP3-F | CCTTCTCCAGGACAGA | 60 | Quantitative RT-PCR of Foxp3 mRNA |
| FOXP3-R | GATCATGGCTGGGTTGT |  |  |
| PD1-F | GCAGTTGAGCTGGCAATCAG | 60 | Quantitative RT-PCR of PD-1 mRNA |
| PD1-R | GTGAAGGTGGCATTTGCTCC |  |  |
| IL-10-F | TGCTGCCTGCTCTTACTGAC | 58 | Quantitative RT-PCR of IL-10 mRNA |
| IL-10-R | GGGGCATCACTTCTACCAGG |  |  |
| IL-17a-F | CCCTCAGACTACCTCAACCG | 60 | Quantitative RT-PCR of IL-17 mRNA |
| IL-17a-R | CAGCTTTCCCTCCGCATTGA |  |  |
| IFNg-F | AGGAACTGGCAAAAGGATGGTG | 60 | Quantitative RT-PCR of IFN-γ mRNA |
| IFNg-R | GCTGGACCTGTGGGTTGTT |  |  |
| DNMT1-F | ACGGAAACCCAAGGAAGAGT | 60 | Quantitative RT-PCR of DNMT1 mRNA |
| DNMT1-R | GCAGCACCACTCTCTGTGT |  |  |
| Tbet-F | GCATGCCAGGGAACCGC | 59 | Quantitative RT-PCR of Tbet mRNA |
| Tbet-R | CATTCACCTCCACGATGTGC |  |  |
| ROR-g-T-F | CTGCAAAGAAGACCCACACCT | 60 | Quantitative RT-PCR of RORγT mRNA |
| ROR-g-T-R | GTGCAGGAGTAGGCCACATTA |  |  |
| GATA3-F | CCACCCCATTACCACCTATCC | 60 | Quantitative RT-PCR of GATA-3 mRNA |
| GATA3-R | CACACACTCCCTGCCTTCTGT |  |  |
| 18S-F | CCGCCGCCATGTCTCTAGT | 58 | Quantitative RT-PCR of 18S rRNA (housekeeping gene) |
| 18S-R | CTTTCCTCAACACCACATGAGC | 58 |  |
| SATB1-F | GAAAGGAGCTTGAACGCAGC | 60 | Quantitative RT-PCR of SATB1 mRNA |
| SATB1-R | CTCTCAGTGGCAAGGGTAGC | 60 |  |
| GATA1-F | GGGACAGGACAGGTCACTAC | 59 | Quantitative RT-PCR of GATA1 mRNA |
| GATA1-R | AGGCATTGCATACCGGATCTC | 59 |  |
| EOS-F | GTGAAAGTAGCGGGGACTCA | 59 | Quantitative RT-PCR of EOS mRNA |
| EOS-R | GTCTGCTCGACTCCTCATCG | 59 |  |
| IRF4-F | AATCCCCATTGAGCCAAGCA | 60 | Quantitative RT-PCR of IRF4 mRNA |
| IRF4-R | CTCGTCGTGGTCAGCTCTTT | 60 |  |
| LEF1-F | CGGGAAGAGCAGGCCAAATA | 59 | Quantitative RT-PCR of LEF1 mRNA |
| LEF1-R | AGCTTCTCTTACCACCTGAAGTC | 59 |  |
